# Computed tomography volumetry of esophageal cancer - the role of semiautomatic assessment

**DOI:** 10.1186/s12880-019-0317-5

**Published:** 2019-02-15

**Authors:** Yi-Hua Zhang, Michael A. Fischer, Henrik Lehmann, Åse Johnsson, Ioannis Rouvelas, Gunnar Herlin, Lars Lundell, Torkel B. Brismar

**Affiliations:** 1 Department of Diagnostic Radiology and Karolinska Institutet, Karolinska University Hospital, CLINTEC, Stockholm, Sweden; 2 Department of Surgery, Centre for Digestive Diseases and Karolinska Institutet, Karolinska University Hospital, CLINTEC, Stockholm, Sweden; 3 Department of Radiology, Institute of Clinical Sciences, The Sahlgrenska Academy at University of Gothenburg, Gothenburg, Sweden; 4 Department of Radiology, Sahlgrenska University Hospital, Gothenburg, Sweden; 5 Division of Medical Imaging and Technology, Department of Clinical Science, Intervention and Technology, Karolinska University Hospital, Huddinge, 141 86 Stockholm, Sweden

**Keywords:** Esophageal cancer, Computed tomography, Tumor volume, Esophageal resection

## Abstract

**Background:**

The clinical and research value of Computed Tomography (CT) volumetry of esophageal cancer tumor size remains controversial. Development in CT technique and image analysis has made CT volumetry less cumbersome and it has gained renewed attention. The aim of this study was to assess esophageal tumor volume by semi-automatic measurements as compared to manual.

**Methods:**

A total of 23 esophageal cancer patients (median age 65, range 51–71), undergoing CT in the portal-venous phase for tumor staging, were retrospectively included between 2007 and 2012. One radiology resident and one consultant radiologist measured the tumor volume by semiautomatic segmentation and manual segmentation. Reproducibility of the respective measurements was assessed by intraclass correlation coefficients (ICC) and by average deviation from mean.

**Results:**

Mean tumor volume was 46 ml (range 5-137 ml) using manual segmentation and 42 ml (range 3-111 ml) using semiautomatic segmentation. Semiautomatic measurement provided better inter-observer agreement than traditional manual segmentation. The ICC was significantly higher for semiautomatic segmentation in comparison to manual segmentation (0.86, 0.56, *p* < 0.01). The average absolute percentage difference from mean was reduced from 24 to 14% (*p* < 0.001) when using semiautomatic segmentation.

**Conclusions:**

Semiautomatic analysis outperforms manual analysis for assessment of esophageal tumor volume, improving reproducibility.

## Background

Despite the overall dismal prognosis of patients with esophageal cancer, therapeutic progress has been made and improvement in effectiveness of therapeutic regimens is emerging [1–3]. At the time of diagnosis, patients with carcinoma of the esophagus often have a locally advanced disease stage with or without distant metastasis [1]. The proportion of patients who can be offered treatment with curative intent is often centered around 25%, a figure which has remained quite stable over time [1, 4–6]. The predominant symptom generated by these tumors is dysphagia and weight loss. Depending on a variety of factors, the obstruction to the passage of food, through the expanding and stricturing tumor area, results in clinically overt symptoms first at a relatively advanced local stage of the disease [7]. In the evaluation of these patients, accurate staging is mandatory and hereby endoscopic ultrasonography, computed tomography (CT) and positron emission tomography (PET) with fluorine 18 fluorodeoxyglucose (FDG) have taken a central role. The main problem with endoscopic ultrasonography is the dependency on the investigator’s level of expertise [8, 9]. Although FDG PET is frequently used in clinical practice, the scientific validity of this technology has to be better defined [10, 11]. Accordingly, in many referral centers, CT remains the investigation of choice, not only for staging but also for the evaluation of the effectiveness of neoadjuvant therapies [12]. In the attempt to describe the extent of the local tumor growth and also when exploring an eventual therapeutic effect of preoperative therapies, assessment of the volume of the tumor might be critical [12, 13]. Attempts have been made to apply this technique both in controlled as well as uncontrolled research protocols [13–16]. Some studies indicate that CT-determined volume of esophageal cancer may add to the assessment of neoadjuvant chemoradiotherapy effects and even add prognostic information [13, 17]. However, at present, there is no established and validated method to monitor esophageal tumor response to treatment.

The aim of the current study was therefore to compare the reproducibility of CT volumetry of esophageal tumors using traditional manual segmentation with more modern semiautomatic segmentation by consultant radiologists and radiologists under training.

## Methods

### Patients

A subset of 23 out of 181 esophageal cancer patients (median age 65 range 51–71, 20 male, 3 female, Table 1) included in a multicenter randomized clinical trial comparing two neoadjuvant regimens during 2007 and 2012 was retrospectively analyzed [18]. The patients had newly diagnosed adenocarcinoma or squamous cell carcinoma and were planned for curative neoadjuvant treatment followed by surgical resection. Tumor histology was verified through histological typing of surgically resected tumor, or multiple endoscopic biopsies if the patient was not applicable for surgical treatment due to disease progression during neoadjuvant treatment. Patients with metastatic diseases or subject to endoscopic stent placement or other treatment prior to the CT scan were excluded. A further inclusion criterion was presence of baseline spiral CT for tumor staging from our clinic before start of neoadjuvant treatment with the presence of scans from both arterial and portal-venous phase and 0.625 mm slices.

**Table 1:** Details about patients (*n* = 23) included for the manual segmentation and semiautomatic measurements

	BMI	Cancer type	TNM stage	Tumor location	Neoadjuvant therapy	Resected
1	22	SCC	T2N1M0	Middle	Chemoradiotherapy	Yes
2	25	AC	T3N1M0	Cardia, SII	Chemoradiotherapy	Yes
3	25	AC	T2N0M0	Distal	Chemoradiotherapy	No
4	24	AC	T3N1M0	Cardia, SII	Chemotherapy	Yes
5	24	AC	T3N1M0	Cardia, SII	Chemotherapy	Yes
6	22	SCC	T3N1M0	Middle	Chemotherapy	Yes
7	22	SCC	T3N1M0	Middle	Chemotherapy	Yes
8	30	AC	T3N1M0	Distal	Chemoradiotherapy	Yes
9	22	AC	T3N0M0	Cardia	Chemoradiotherapy	No
10	27	SCC	T3N1M0	Middle	Chemoradiotherapy	Yes
11	24	AC	T3N1M0	Distal	Chemotherapy	Yes
12	32	SCC	T3N1M0	Distal	Chemotherapy	Yes
13	33	AC	T3N1M0	Cardia, SII	Chemoradiotherapy	Yes
14	25	AC	T2N0M0	Cardia, SI	Chemotherapy	Yes
15	28	AC	T3N1M0	Cardia, SII	Chemotherapy	Yes
16	23	SCC	T2N1M0	Middle	Chemoradiotherapy	Yes
17	30	AC	T3N0M0	Cardia, SII	Chemoradiotherapy	Yes
18	21	SCC	T3N1M0	Distal	Chemotherapy	No
19	22	SCC	T3N1MX	Distal	Chemoradiotherapy	Yes
20	34	AC	T2N0M0	Cardia, SII	Chemotherapy	Yes
21	26	AC	T3N0M0	Cardia, SII	Chemoradiotherapy	Yes
22	20	AC	T3N1M0	Cardia, SII	Chemoradiotherapy	Yes
23	23	AC	T3N0M0	Cardia, SII	Chemoradiotherapy	Yes

*AC* = Adenocarcinoma, *SCC* Squamous cell carcinoma, *SI* = Siewert I, *SII* = Siewert II

Ethical approval for the study was granted by the regional ethical review board in Stockholm. Approval number: DNR 2008/403–32. Written informed consent was obtained.

### CT imaging acquisition parameters

The patients underwent multi-slice CT of the thorax using multislice CT (GE Lightspeed VCT (GE Healthcare, WI, USA) or Siemens Somatom Definition Flash (Siemens AG, Erlangen, Germany). All examinations were performed at 120 kV after intravenous contrast injection of Iomeron 400 mg I/ml (Bracco, Milan Italy) in both arterial and portal phase. The tube current was automatically modulated. The dosage of contrast media was 750 mg I/kg or 1000 mg I/kg. Slice thickness was 0.625 mm. The field of view was adjusted for patient size.

### Comparison of manual and semiautomatic segmentation

A second year resident in radiology and a consultant radiologist with 25 years of experience independently measured the tumor volume of 23 patients with esophageal cancer (middle and distal third part) by manual and semiautomatic segmentation. These patients were under baseline evaluation for curative resections for esophageal cancer after induction chemo or radio-chemo therapy.

The segmentation was performed using a dedicated workstation with GE AW 4.0 (GE Healthcare, WI, USA).

**Fig. 1:**
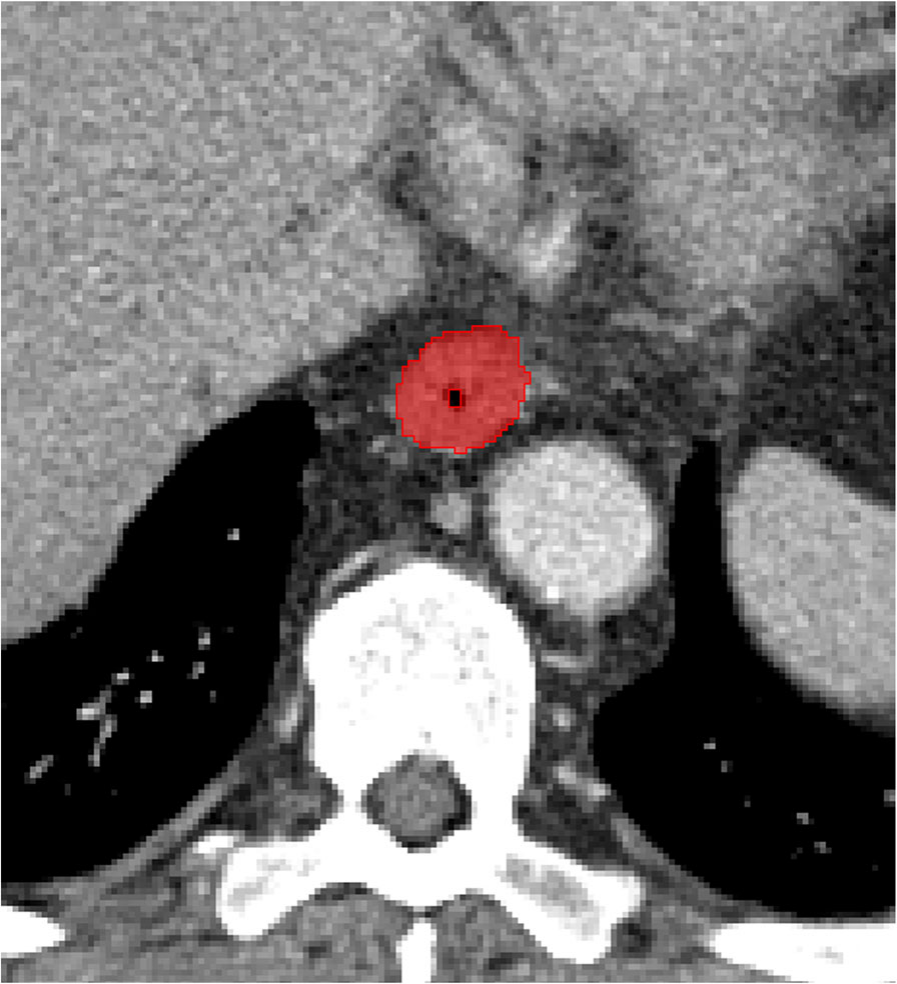
A transaxial image of esophagus showing the delineation of the tumor using manual segmentation

Images were first reformatted to 2.5 mm and displayed as average intensity projections. CT window level settings were at the discretion of the observer. Only transaxial images were available for the observers. For the semiautomated segmentation, the first and last slice containing the primary esophageal tumor, and slices where major morphologic changes occurred, were delineated manually using a mouse controlled cursor (Fig. 1). The rest of the tumor was then first interpolated by the software and the resulting volume of interest was reviewed by the radiologist and manually adjusted by adding or removing included tumor area for each slice where disagreement with the software interpolated selection occurred. The lower and higher threshold of voxels included in the volume of interest was set to 0 and 1000 Hounsfield units respectively in order to exclude air and include all esophageal tumor tissue. The cross sectional areas of all slices were multiplied by the slice thicknesses and the total volume was calculated by summation of these volumes. The measurement of the tumors was done in both arterial and venous phase for each patient, resulting in two measurements of volume per tumor per observer.

The manual segmentation was done by the same observers at least three months after the measurement using semiautomated segmentation to reduce the effects of recall of the previous semiautomated segmentation. The tumor was manually delineated on transaxial images on every slice containing the primary esophageal tumor and tumor volume was calculated by multiplying cross sectional areas of all slices by the slice thickness and summation of the resulting volumes.

### Statistical analyses

Data are presented as mean values (95% confidence interval of the mean, CI). Statistical significance was defined at a level of *p* < 0.05. Intraclass correlation coefficients (ICC) were calculated for intra and inter-observer measurements. The significance of difference in correlation was tested using a Fisher r to z calculation. To further characterize the level of observer agreement Bland-Altman plots were used to graphically visualize the level of agreement. Upper and lower limits of agreement were calculated and incorporated into the plots [19]. Observer measurement accuracy was also assessed by calculating the average absolute difference from mean for each tumor volume measurement. Comparison of tumor volume between the arterial phase and the portovenous phase was used to assess intraobserver variability of measurement.

Statistical analysis was done using R 3.4.3 (R Foundation, Vienna, Austria).

### Availability of data and material

The datasets generated and/or analyzed during the current study are not publicly available but are available from the corresponding author on reasonable request.

## Results

All tumors were detected by both observers for all included patients. Mean tumor volume when merging arterial and portovenous measurements was 46 ml (range 5-137 ml) using manual segmentation and 42 ml (range 3-111 ml) using semiautomatic segmentation, (*p* = 0.30). No significant differences in volume were observed between adenocarcinoma and squamous cell carcinoma. All measured volumes are shown in Table 2.

**Table 2:** Measured primary esophageal tumor volume for all included patients by radiology resident and radiology consultant using manual and semiautomatic segmentation. All volumes measured in milliliters (ml)

	Manual				Semiautomatic			
	Resident		Consultant		Resident		Consultant	
	Arterial	Venous	Arterial	Venous	Arterial	Venous	Arterial	Venous
1	15	14	5	5	33	31	3	3
2	42	44	13	8	36	31	24	28
3	36	44	5	6	23	26	25	25
4	44	50	16	15	28	21	26	25
5	57	52	30	33	48	59	29	35
6	46	54	43	31	39	33	33	36
7	105	99	62	63	70	83	68	74
8	135	125	81	70	95	101	69	68
9	51	66	21	23	34	42	19	26
10	65	56	23	35	47	40	41	40
11	137	132	73	78	111	110	92	104
12	73	81	58	59	59	62	59	54
13	66	69	45	48	50	50	40	38
14	27	35	17	17	19	22	17	16
15	49	64	36	36	47	55	28	31
16	32	30	24	24	18	24	20	25
17	56	46	35	32	44	34	38	38
18	59	44	36	32	77	77	80	75
19	51	57	42	44	39	47	37	41
20	48	48	11	12	28	36	20	19
21	25	42	11	12	13	19	10	14
22	41	38	24	29	16	15	20	28
23	69	75	51	47	55	62	52	53
Mean (CI 95%)	58 (44–71)	59 (47–72)	33 (24–42)	33 (24–42)	45 (34–55)	47 (36–58)	37 (27–47)	39 (29–49)

### Intraobserver variability of tumor assessment at CT

**Fig. 2:**
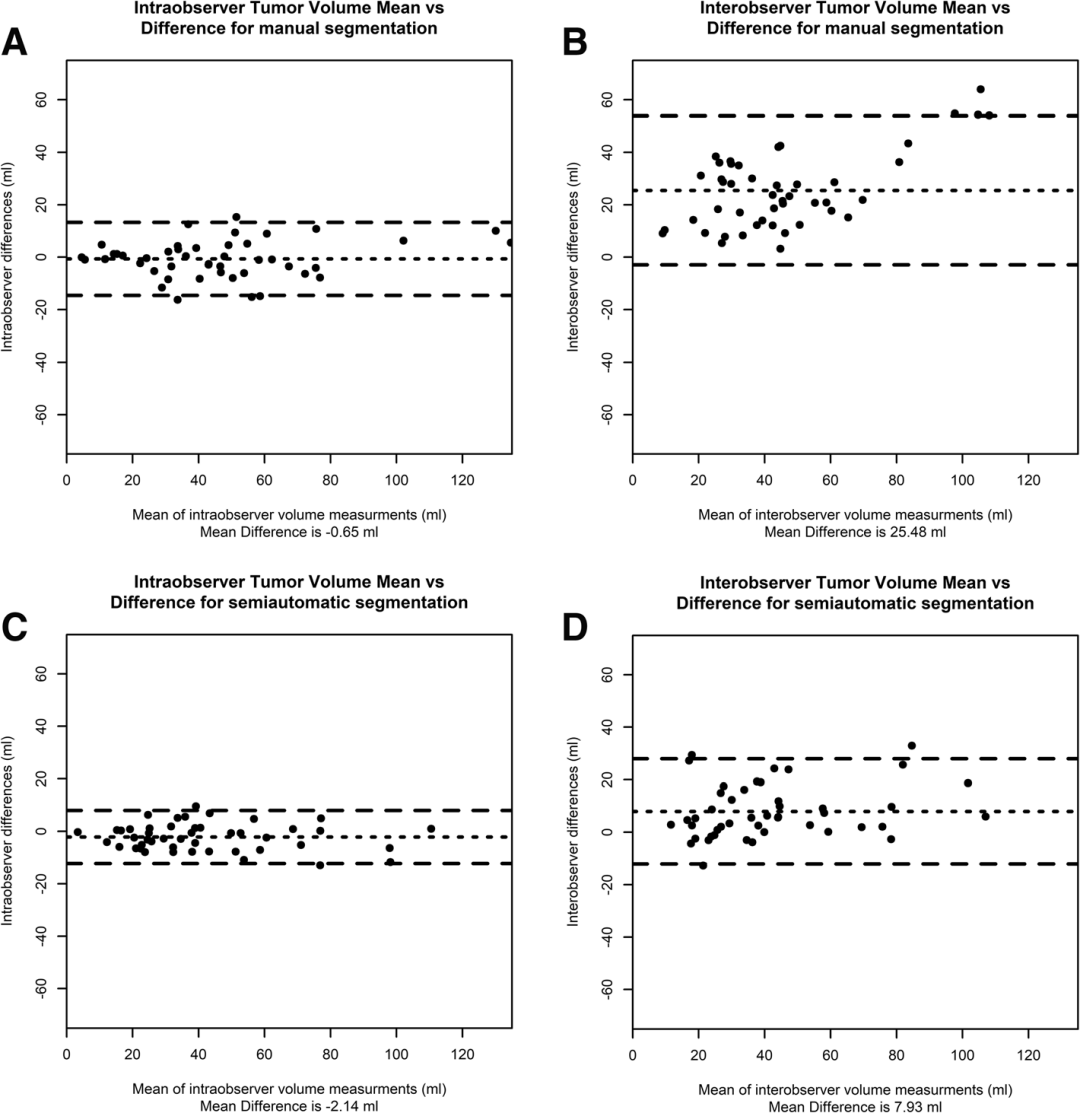
**a**-**d** Bland-Altman plots for intraobserver and interobserver differences during tumor volume measurement using manual and semiautomatic segmentation. The difference of tumor volume is plotted against the mean. The dashed lines are calculated as ±1.96SD and show upper and lower limits of agreement. The dotted line shows the mean difference

No statistically significant difference of mean tumor volume was observed between arterial and portovenous volume measurements for both manual and semiautomatic methods for both observers. Comparison of arterial tumor volume with portovenous volume resulted in excellent intraobserver agreement with ICC of 0.97 for both manual and semiautomatic segmentation. Bland-Altman plots (Fig. 2a, c) show low variability in comparison to interobserver variability.

### Interobserver variability of tumor assessment at CT

Interobserver ICC was significantly higher for semiautomatic segmentation in comparison to manual segmentation (0.86 versus 0.56, *p* < 0.01). Bland-Altman plots (Fig. 2b, d) show slightly narrower limits for semiautomatic segmentation in comparison to manual segmentation, (40.1 ml versus 56.8 ml). Significantly higher ICC was observed after semiautomatic segmentation compared to manual segmentation for measurements of adenocarcinoma (0.86 versus 0.54, *p* < 0.01) but not for squamous cell carcinoma (0.88 versus 0.63, *p* = 0.052). No significant differences in ICC between adenocarcinoma and squamous cell carcinoma were detected when sub analyzing the manual segmentation or semiautomatic segmentation group.

**Fig. 3:**
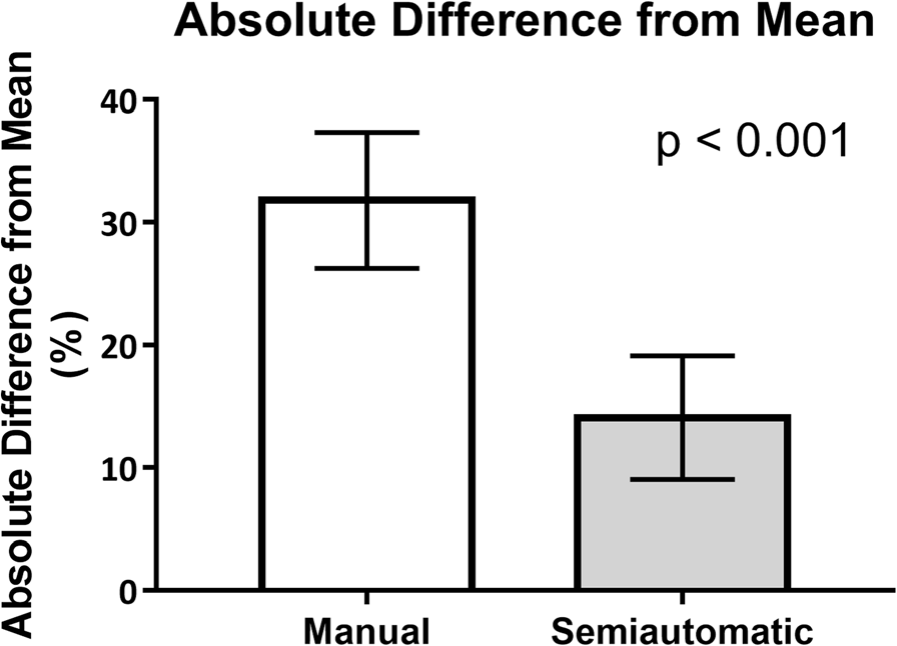
Mean absolute differences from the mean measured volume of the esophageal tumor by use of semiautomatic segmentation and manual segmentation, respectively. Bars show 95% confidence interval

The average absolute percentage difference from mean tumor volume was significantly lower when using semiautomatic segmentation (14%, CI:9–19%) than when using manual segmentation (32%, CI: 26–37%, *p* < 0.001, Fig. 3). The percentage difference was significantly lower for squamous cell carcinoma compared to adenocarcinoma (23, 36%, *p* < 0.05) when using manual segmentation. This difference was not observed for semiautomatic segmentation.

## Discussion

In comparison to manual segmentation, the use of semiautomatic segmentation resulted in a higher interobserver agreement and a lower average absolute percentage difference from mean volume when comparing esophageal tumors volumes segmented by consultant and resident radiologists.

The clinical and research values of CT volumetry at esophageal cancer management are controversial [8, 20, 21]. One possible reason behind this is the fact that CT technology and volumetry techniques used have not been sufficiently addressed and therefore may be suboptimal. For instance, small difference in image contrast between the tumor and the normal esophagus tissues may result in substantial variability in the final calculations. This variation is probably user and experience dependent. In this study, it was observed that the resident, but not the experienced radiologist, had a greater variation when manually delineating adenocarcinoma than squamous carcinoma. This might be explained by small differences in tumor texture [22], which are probably too small to allow the radiologist to diagnose tumor subtype. However, we have recently shown that computerized image analysis, so called CT morphometry, can distinguish between esophageal adenocarcinoma and squamous cell carcinoma [22]. Those small differences in texture might aid the experienced radiologist to better delineate adenocarcinoma but might be too small to the resident to discern. This would explain why the observed difference in tumor volume assessment variation between the resident and consultant radiologist differed between tumor subtype.

One study reported great variations in repeated measurements done by the same observer and also between expert radiologists using the manual segmentation approach [23]. In our study, we observed that by using a semiautomatic segmentation technique, we could significantly reduce this variation to the level of excellent agreement and making the measurements independent on the level of experience of the assessor. A recent study comparing different semiautomatic segmentation software has shown similar excellent intra- and inter observer agreement [24].

Different criteria have been used for the morphological evaluation of esophageal tumors, ranging from bi-dimensional measurement of tumor lesions according to the WHO criteria [25] to the thickness of the esophageal wall [20, 26] or assessment of the volume by use of stereology [27]. In our study, we used the summation-of-area method described by Breiman [28]. This is a simple method which does not require any sophisticated mathematical formulas and has been mainly used to assess the volume of solid organs such as the liver and spleen and also tumor masses e.g. head and neck and kidney.

The observed discrepancy between the readers when evaluating individual tumors can be attributed to several contributing factors. The main individual factor was probably the small difference in image contrast between the tumor and the normal esophagus tissue. This resulted in difficulties in defining the respective cranial and caudal borders of the tumors, especially in tumors located close to or at the gastro-esophageal junction. In addition, the current CT scans consisted only of axial images whereupon no multiplanar reformations were available to the readers. Thinner collimation and coronal and sagittal reformations may add to a better delineation of the cranial and caudal borders of the tumors. Other possible ways to improve the definition of the tumor borders can be to use positive or negative oral contrast media just prior to the CT examination and by the aid of antispasmodic agents [14, 29]. On the other hand, the introduction of a specific, more complex CT protocol for the study of the esophagus might be difficult to implement into clinical routine practice outside tertiary referral centers, where the esophageal tumors are examined with standard CT examinations in the N and M-staging process.

Other methods than CT can add to the armamentarium of methods allowing tumor volume detection and assessment of changes therein. PET-CT imaging with 18 FDG has recently been shown to offer advantages in monitoring the response to neoadjuvant treatment of esophageal cancer by measuring the metabolic/volume activity [21, 30, 31]. However, uncertainties regarding which thresholds of standardized uptake value (SUV) during the delineation of tumor remain as a source of variability in previous studies and there is currently no standardized protocol in use. Analysis of apparent diffusion coefficient (ADC) using diffusion-weighted magnetic resonance imaging (DWI-MRI) has been shown to correlate with histological tumor response and tumor staging [32–34]. The need to segment tumor volume in order to calculate ADC highlights the importance of reducing the interobserver variation of the tumor segmentation.

Recent developments of computing power have enabled quantification of textural parameters of tumor volumes segmented from both CT and PET images, which has been shown to correlate with overall survival and treatment response in several studies [35–37], but not in all [22]. However, these methods are sensitive to segmentation errors and accurate segmentation methods are needed in order to ensure comparable results between studies [38, 39].

There are some limitations burdening this study. The number of patients was relatively small and there were only two readers, which exposes the outcome to the risk of the random effect of single outliers. A further sub analysis of differences between segmentation methods depending on histological type might have not shown significance due to lack of enough patients per group (*n* = 15 versus *n* = 8). The patients were also scanned on two different scanners. However, this should not impact the comparison between semiautomatic and manual segmentation.

## Conclusions

In conclusion, when compared to manual segmentation, application of semiautomatic CT volumetry of esophageal tumors obtained by using modern CT technology, reduces the interobserver variability, regardless of the observer’s experience.

## Abbreviations

CI: 95% confidence interval of the mean
CT: Computed tomography
DWI-MRI: Diffusion-weighted magnetic resonance imaging
FDG: Fluorine 18 fluorodeoxyglucose
ICC: Intraclass correlation coefficients
PET: Positron emission tomography
SUV: Standardized uptake value

## References

[1] Rouvelas I, Zeng W, Lindblad M, Viklund P, Ye W, Lagergren J (2005). Survival after surgery for oesophageal cancer: a population-based study. Lancet Oncol..

[2] Wolf MC, Stahl M, Krause BJ, Bonavina L, Bruns C, Belka C (2011). Curative treatment of oesophageal carcinoma: current options and future developments. Radiat Oncol.

[3] Nagaraja V, Cox MR, Eslick GD (2014). Safety and efficacy of esophageal stents preceding or during neoadjuvant chemotherapy for esophageal cancer: a systematic review and meta-analysis. J Gastrointest Oncol.

[4] Vallböhmer D, Brabender J, Grimminger P, Schröder W, Hölscher AH (2011). Predicting response to neoadjuvant therapy in esophageal cancer. Expert Rev Anticancer Ther.

[5] Low DE (2011). Update on staging and surgical treatment options for esophageal cancer. J Gastrointest Surg.

[6] Mariette C, Piessen G, Triboulet J-P (2007). Therapeutic strategies in oesophageal carcinoma: role of surgery and other modalities. Lancet Oncol..

[7] Liedman BL, Bennegård K, Olbe LC, Lundell LR (1995). Predictors of postoperative morbidity and mortality after surgery for gastro-oesophageal carcinomas. Eur J Surg.

[8] Cerfolio RJ, Bryant AS, Ohja B, Bartolucci AA, Eloubeidi MA (2005). The accuracy of endoscopic ultrasonography with fine-needle aspiration, integrated positron emission tomography with computed tomography, and computed tomography in restaging patients with esophageal cancer after neoadjuvant chemoradiotherapy. J Thorac Cardiovasc Surg.

[9] Choi J, Kim SG, Kim JS, Jung HC, Song IS (2010). Comparison of endoscopic ultrasonography (EUS), positron emission tomography (PET), and computed tomography (CT) in the preoperative locoregional staging of resectable esophageal cancer. Surg Endosc.

[10] Lordick F, Ott K, Krause B-J, Weber WA, Becker K, Stein HJ (2007). PET to assess early metabolic response and to guide treatment of adenocarcinoma of the oesophagogastric junction: the MUNICON phase II trial. Lancet Oncol.

[11] Lordick F, Ott K, Krause BJ (2010). New trends for staging and therapy for localized gastroesophageal cancer: the role of PET. Ann Oncol.

[12] Boone J, Livestro DP, Elias SG, Borel Rinkes IHM, van Hillegersberg R (2009). International survey on esophageal cancer: part II staging and neoadjuvant therapy. Dis Esophagus.

[13] Beer AJ, Wieder HA, Lordick F, Ott K, Fischer M, Becker K (2006). Adenocarcinomas of esophagogastric junction: multi-detector row CT to evaluate early response to neoadjuvant chemotherapy. Radiology.

[14] Griffith JF, Chan AC, Chow LT, Leung SF, Lam YH, Liang EY (1999). Assessing chemotherapy response of squamous cell oesophageal carcinoma with spiral CT. Br J Radiol.

[15] Sohaib SA, Turner B, Hanson JA, Farquharson M, Oliver RT, Reznek RH (2000). CT assessment of tumour response to treatment: comparison of linear, cross-sectional and volumetric measures of tumour size. Br J Radiol.

[16] van Heijl M, Phoa SSKS, van Berge Henegouwen MI, Omloo JMT, Mearadji BM, Sloof GW (2011). Accuracy and reproducibility of 3D-CT measurements for early response assessment of chemoradiotherapy in patients with oesophageal cancer. Eur J Surg Oncol.

[17] Créhange G, Bosset M, Lorchel F, Fabrice L, Buffet-Miny J, Dumas JL (2006). Tumor volume as outcome determinant in patients treated with chemoradiation for locally advanced esophageal cancer. Am J Clin Oncol.

[18] Klevebro F, von Alexandersson Döbeln G, Wang N, Johnsen G, Jacobsen AB, Friesland S (2016). A randomized clinical trial of neoadjuvant chemotherapy versus neoadjuvant chemoradiotherapy for cancer of the oesophagus or gastro-oesophageal junction. Ann Oncol.

[19] Bland JM, Altman DG (1986). Statistical methods for assessing agreement between two methods of clinical measurement. Lancet.

[20] Swisher SG, Maish M, Erasmus JJ, Correa AM, Ajani JA, Bresalier R (2004). Utility of PET, CT, and EUS to identify pathologic responders in esophageal cancer. Ann Thorac Surg.

[21] Tamandl D, Gore RM, Fueger B, Kinsperger P, Hejna M, Paireder M (2016). Change in volume parameters induced by neoadjuvant chemotherapy provide accurate prediction of overall survival after resection in patients with oesophageal cancer. Eur Radiol.

[22] Zhang Y-H, Herlin G, Rouvelas I, Nilsson M, Lundell L, Brismar TB. Texture analysis of computed tomography data using morphologic and metabolic delineation of esophageal cancer-relation to tumor type and neoadjuvant therapy response. Dis Esophagus. 2018. Epub ahead of print. 10.1093/dote/doy096 30295752

[23] Hermans R, Feron M, Bellon E, Dupont P, Van den Bogaert W, Baert AL (1998). Laryngeal tumor volume measurements determined with CT: a study on intra- and interobserver variability. Int J Radiat Oncol Biol Phys.

[24] MacKeith SAC, Das T, Graves M, Patterson A, Donnelly N, Mannion R (2018). A comparison of repeatability and usability of semi-automated volume segmentation tools for measurement of vestibular schwannomas. Otol Neurotol.

[25] Kroep JR, Van Groeningen CJ, Cuesta MA, Craanen ME, Hoekstra OS, Comans EFI (2003). Positron emission tomography using 2-deoxy-2-[18F]-fluoro-D-glucose for response monitoring in locally advanced gastroesophageal cancer; a comparison of different analytical methods. Mol Imaging Biol.

[26] Jones DR, Parker LA, Detterbeck FC, Egan TM (1999). Inadequacy of computed tomography in assessing patients with esophageal carcinoma after induction chemoradiotherapy. Cancer.

[27] Okur A, Kantarci M, Akgun M, Alper F, Cayir K, Koc M (2005). Unbiased estimation of tumor regression rates during chemoradiotherapy for esophageal carcinoma using CT and stereology. Dis Esophagus.

[28] Breiman RS, Beck JW, Korobkin M, Glenny R, Akwari OE, Heaston DK (1982). Volume determinations using computed tomography. AJR Am J Roentgenol.

[29] Liang EY, Chan A, Chung SC, Metreweli C (1996). Short communication: oesophageal tumour volume measurement using spiral CT. Br J Radiol.

[30] Larson SM, Schoder H, Yeung H (2004). Positron emission tomography/computerized tomography functional imaging of esophageal and colorectal cancer. Cancer J.

[31] Sloof GW (2006). Response monitoring of neoadjuvant therapy using CT, EUS, and FDG-PET. Best Pract Res Clin Gastroenterol.

[32] van Rossum PSN, van Lier ALHMW, van Vulpen M, Reerink O, Lagendijk JJW, Lin SH (2015). Diffusion-weighted magnetic resonance imaging for the prediction of pathologic response to neoadjuvant chemoradiotherapy in esophageal cancer. Radiother Oncol.

[33] De Cobelli F, Giganti F, Orsenigo E, Cellina M, Esposito A, Agostini G (2013). Apparent diffusion coefficient modifications in assessing gastro-oesophageal cancer response to neoadjuvant treatment: comparison with tumour regression grade at histology. Eur Radiol.

[34] Huang Y-C, Chen T-W, Zhang X-M, Zeng N-L, Li R, Tang Y-L (2018). Intravoxel incoherent motion diffusion-weighted imaging of resectable oesophageal squamous cell carcinoma: association with tumour stage. Br J Radiol.

[35] Yip C, Landau D, Kozarski R, Ganeshan B, Thomas R, Michaelidou A (2014). Primary esophageal cancer: heterogeneity as potential prognostic biomarker in patients treated with definitive chemotherapy and radiation therapy. Radiology.

[36] Yip C, Davnall F, Kozarski R, Landau DB, GJR C, Ross P, et al. Assessment of changes in tumor heterogeneity following neoadjuvant chemotherapy in primary esophageal cancer. Dis Esophagus. 2015;28:172–9. 10.1111/dote.12170 24460831

[37] Ganeshan B, Skogen K, Pressney I, Coutroubis D, Miles K (2012). Tumour heterogeneity in oesophageal cancer assessed by CT texture analysis: preliminary evidence of an association with tumour metabolism, stage, and survival. Clin Radiol.

[38] Hatt M, Tixier F, Pierce L, Kinahan PE, Le Rest CC, Visvikis D (2017). Characterization of PET/CT images using texture analysis: the past, the present… any future?. Eur J Nucl Med Mol Imaging.

[39] Doumou G, Siddique M, Tsoumpas C, Goh V, Cook GJ (2015). The precision of textural analysis in (18)F-FDG-PET scans of oesophageal cancer. Eur Radiol.

